# Impact of social determinants on antiretroviral therapy access and outcomes entering the era of universal treatment for people living with HIV in Italy

**DOI:** 10.1186/s12889-018-5804-z

**Published:** 2018-07-13

**Authors:** Annalisa Saracino, Mauro Zaccarelli, Patrizia Lorenzini, Alessandra Bandera, Giulia Marchetti, Francesco Castelli, Andrea Gori, Enrico Girardi, Cristina Mussini, Paolo Bonfanti, Adriana Ammassari, Antonella d’Arminio Monforte, for the Icona Foundation Study Group, M. Moroni, M. Andreoni, G. Angarano, A. Antinori, A. d’Arminio Monforte, F. Castelli, R. Cauda, G. Di Perri, M. Galli, R. Iardino, G. Ippolito, A. Lazzarin, C. F. Perno, F. von Schloesser, P. Viale, A. d’Arminio Monforte, A. Antinori, A. Castagna, F. Ceccherini-Silberstein, A. Cozzi-Lepri, E. Girardi, S. Lo Caputo, C. Mussini, M. Puoti, M. Andreoni, A. Ammassari, A. Antinori, C. Balotta, P. Bonfanti, S. Bonora, M. Borderi, M. R. Capobianchi, A. Castagna, F. Ceccherini-Silberstein, A. Cingolani, P. Cinque, A. Cozzi-Lepri, A. d’Arminio Monforte, A. De Luca, A. Di Biagio, E. Girardi, N. Gianotti, A. Gori, G. Guaraldi, G. Lapadula, M. Lichtner, S. Lo Caputo, G. Madeddu, F. Maggiolo, G. Marchetti, S. Marcotullio, L. Monno, C. Mussini, M. Puoti, E. Quiros Roldan, S. Rusconi, A. Saracino, A. Cozzi-Lepri, P. Cicconi, I. Fanti, L. Galli, P. Lorenzini, M. Shanyinda, A. Tavelli, A. Giacometti, A. Costantini, S. Mazzoccato, G. Angarano, L. Monno, C. Santoro, F. Maggiolo, C. Suardi, P. Viale, E. Vanino, G. Verucchi, F. Castelli, E. Quiros Roldan, C. Minardi, T. Quirino, C. Abeli, P. E. Manconi, P. Piano, J. Vecchiet, K. Falasca, L. Sighinolfi, D. Segala, F. Mazzotta, S. Lo Caputo, G. Cassola, C. Viscoli, A. Alessandrini, R. Piscopo, G. Mazzarello, C. Mastroianni, V. Belvisi, P. Bonfanti, I. Caramma, A. Chiodera, A. P. Castelli, M. Galli, A. Lazzarin, G. Rizzardini, M. Puoti, A. d’Arminio Monforte, A. L. Ridolfo, R. Piolini, A. Castagna, S. Salpietro, L. Carenzi, M. C. Moioli, C. Tincati, G. Marchetti, C. Mussini, C. Puzzolante, A. Gori, G. Lapadula, N. Abrescia, A. Chirianni, G. Borgia, M. G. Guida, M. Gargiulo, I. Gentile, R. Orlando, F. Baldelli, D. Francisci, G. Parruti, T. Ursini, G. Magnani, M. A. Ursitti, R. Cauda, M. Andreoni, A. Antinori, V. Vullo, A. Cingolani, A. d’Avino, L. Gallo, E. Nicastri, R. Acinapura, M. Capozzi, R. Libertone, G. Tebano, M. Zaccarelli, F. Viviani, L. Sasset, M. S. Mura, G. Madeddu, A. De Luca, B. Rossetti, P. Caramello, G. Di Perri, G. C. Orofino, S. Bonora, M. Sciandra, M. Bassetti, A. Londero, G. Pellizzer, V. Manfrin

**Affiliations:** 1 Clinic of Infectious Diseases, University of Bari, Piazza G. Cesare, 11 –, 70124 Bari, Italy; 2 Clinical Department, National Institute for Infectious Diseases “Lazzaro Spallanzani” IRCCS, Rome, Italy; 3 Clinic of Infectious Diseases, “San Gerardo” Hospital - University of Milano-Bicocca, Monza, Italy; 4 Clinic of Infectious Diseases, San Paolo Hospital, University of Milan, Milan, Italy; 5 University Division of Infectious and Tropical Diseases, University of Brescia and Spedali Civili General Hospital, Brescia, Italy; 6 Department of Epidemiology, National Institute for Infectious Diseases “L. Spallanzani,” IRCCS, Rome, Italy; 7 Clinic of Infectious Diseases, University of Modena and Reggio Emilia, Modena, Italy; 8 Unit of Infectious Diseases, ASST Lecco, “A. Manzoni” Hospital, Lecco, Italy; 9 Infectious Diseases Unit, Department of Internal Medicine, IRCCS Ca’ Granda, Ospedale Maggiore Policlinico, Milan, Italy

**Keywords:** Social determinants, HIV, Antiretroviral therapy, ICONA

## Abstract

**Background:**

Social determinants are known to be a driving force of health inequalities, even in high income countries. Aim of our study was to determine if these factors can limit antiretroviral therapy (ART) access, outcome and retention in care of people living with HIV (PLHIV) in Italy.

**Methods:**

All ART naïve HIV+ patients (pts) of Italian nationality enrolled in the ICONA Cohort from 2002 to 2016 were included. The association of socio-demographic characteristics (age, sex, risk factor for HIV infection, educational level, occupational status and residency area) with time to: ART initiation (from the first positive anti-HIV test), ART regimen discontinuation, and first HIV-RNA < 50 cp/mL, were evaluated by Cox regression analysis, Kaplan Meier method and log-rank test.

**Results:**

A total of 8023 HIV+ pts (82% males, median age at first pos anti-HIV test 36 years, IQR: 29–44) were included: 6214 (77.5%) started ART during the study period. Women, people who inject drugs (PWID) and residents in Southern Italy presented the lowest levels of education and the highest rate of unemployment compared to other groups. Females, pts aged > 50 yrs., unemployed vs employed, and people with lower educational levels presented the lowest CD4 count at ART initiation compared to other groups. The overall median time to ART initiation was 0.6 years (yrs) (IQR 0.1–3.7), with a significant decrease over time [2002–2006 = 3.3 yrs. (0.2–9.4); 2007–2011 = 1.0 yrs. (0.1–3.9); 2012–2016 = 0.2 yrs. (0.1–2.1), *p* < 0.001]. By multivariate analysis, females (*p* < 0.01) and PWID (*p* < 0.001), presented a longer time to ART initiation, while older people (*p* < 0.001), people with higher educational levels (*p* < 0.001), unemployed (*p* = 0.02) and students (*p* < 0.001) were more likely to initiate ART. Moreover, PWID, unemployed vs stable employed, and pts. with lower educational levels showed a lower 1-year probability of achieving HIV-RNA suppression, while females, older patients, men who have sex with men (MSM), unemployed had higher 1-year risk of first-line ART discontinuation.

**Conclusions:**

Despite median time to ART start decreased from 2002 to 2016, socio-demographic factors still contribute to disparities in ART initiation, outcome and durability.

## Background

Antiretroviral therapy (ART) is now recommended worldwide for all people living with HIV (PLHIV), regardless of their CD4 cell count [1]. However, barriers to ART access for PLHIV do exist, with important differences among countries, especially according to socio-economic levels and organization models of national healthcare systems [2]. These disparities can affect the probability of reaching the “90–90-90” target fixed by UNAIDS for 2030 (90% diagnosed individuals of all PLHIV, 90% treated patients, and 90% PLHIV achieving viral suppression) [3].

Even in high income countries, social determinants are known to be a driving force of health inequalities [4]. In the setting of HIV infection, demographic and socioeconomic factors can limit the access to testing, treatment, and retention in care and, consequently, reduce survival of PLHIV [5]. Entering the era of universal ART for all HIV people living with HIV, we can hypothesize that these factors could have an even greater impact on disparities in ART access compared to past years.

In 2015, the proportion of people newly diagnosed with HIV and presenting with < 200 cell/μL CD4 was 36.6% in Italy, whereas proportion of people with CD4 < 350 cell/μL was 54.5% [6]. In a previous study from the ICONA (Italian Cohort Naïve Antiretrovirals) cohort, among socio-demographic factors, migrant status was already demonstrated to be associated with barriers to ART initiation and with increased risk of treatment failure compared to natives [7]. However, other social or behavioral determinants, including gender [8, 9], poor education [10, 11], unemployment [12], social exclusion within special population groups, e.g. people who inject drugs (PWID), need to be considered [13].

Moreover, also in people accessing ART and adhering to therapy, lifestyle differences can affect chances to respond to ART, occurrence of side effects and co-morbidities in PLHIV [14].

Aim of our study was to determine if social determinants influence ART access, outcome and retention in care of PLHIV in spite of a context of public healthcare. The knowledge of factors which define the “social vulnerability” can allow to allocate resources and plan for interventions in order to reduce disparities which impact survival of persons living with HIV.

## Methods

The ICONA Foundation Study is an observational cohort of people living with HIV who are antiretroviral naïve at the time of enrolment. This cohort was set up in January 1997 and currently consists of over 14,000 patients from 50 Italian infectious disease reference units. Demographic and socio-behavioral data, initiation and discontinuation dates of each antiretroviral drug, HIV-viral load and CD4 cell count every 3–6 months, AIDS defining diseases according to Centers for Disease Control and Prevention (CDC) criteria as well as non-HIV related diseases and death are recorded for all enrolled participants. Further details are available at http://www.fondazioneicona.org/.

In the present study, all ART naïve PLHIV of Italian nationality enrolled in the ICONA Cohort from 2002 to 2016 were included. The following socio-demographic characteristics were retrieved and analyzed for their potential association with the study endpoints: age, gender (male, female), risk factor for HIV infection [heterosexuals (HS), men who have sex with men (MSM), people who inject drugs (PWID)], educational level, occupational status and residency area. Taking into account the structure of the Italian education system, and according to the UNESCO/International Standard Classification of Education standard classification (ISCED) [15], the educational level was categorized into four categories: primary school (corresponding to ISCED 0); junior secondary school (ISCED 1); high secondary school (ISCED 2–3); tertiary school (university and similar) (ISCED ≥4). The occupational status was classified into seven categories as follows: full-time worker, temporary employed, self employed, unemployed, student, retired, housewife (defined as women only managing household affairs without having paid employment). The residency area was categorized in Northern, Central and Southern Italy.

Patients who did not attend visits for ≥18 months were defined as lost to follow-up.

### Statistical analysis

The study time was divided into 3–5 years periods: 2002–2006; 2007–2011; 2012–2016, in order to compare different ART treatment period, according to guidelines changes and progressive availability of new antiretroviral drugs. Patients’ characteristics were compared according to time period using Chi square test for trend for categorical variables and Kruskal Wallis test for continuous parameters.

Three different end-points were evaluated:

- probability of ART initiation from the first positive anti-HIV test;
- virological response defined as first HIV-RNA < 50 cp/mL after therapy start;
- first ART regimen discontinuation for any causes.

For the evaluation of the endpoints (b) and (c) only patients with ≥1 year of follow-up were included.

The association of socio-demographic characteristics (age, sex, risk factor for HIV infection, educational level, occupational status and residency area, smoking habit) and clinical parameters (CDC stage, CD4 and HIV RNA value and pregnancy) with three end-points were evaluated by Cox regression analysis. 1-year probability was evaluated by Kaplan Meier method and log-rank test was used to compare survival curves.

## Results

### Baseline patient characteristics

A total of 8023 HIV-positive patients (82% males, median age at first positive anti-HIV test: 36 years, IQR: 29–44) were included in the ICONA observational database in the period 2002–2016, whose characteristics are shown in Table 1**,** overall and according to the three different study periods. Median age at time of enrolment, proportion of males and MSM increased over time. Moreover, a growing number of people with high secondary and tertiary level of education but a decreasing number of full employed workers was observed in last period (2012–2016). The percentage of pre-treatment AIDS events and very late presenter patients (< 200 CD4/μl) decreased passing from the first to the last period from 13 and 26% to 7 and 17%, respectively.

**Table 1:** Socio-demographic characteristics of patients according to years of starting cART or year of last observation

	Overall *N* = 8023	2002–2006 *N* = 1543	2007–2011 *N* = 2166	2012–2016 *N* = 4314	*p*
Gender, n (%)					
Female	1434 (17.9%)	453 (29.4%)	368 (17.0%)	613 (14.2%)	< 0.001
Male	6589 (82.1%)	1090 (70.6%)	1798 (83.0%)	3701 (85.8%)	
Age at first positive anti-HIV test, n (%)					
< 30	2582 (32.2%)	682 (44.2%)	637 (29.5%)	1263 (29.3%)	< 0.001
30–40	2646 (33.0%)	521 (33.8%)	742 (34.4%)	1383 (32.1%)	
40–50	1709 (21.3%)	221 (14.3%)	475 (22.0%)	1013 (23.5%)	
> 50	1079 (13.5%)	119 (7.7%)	305 (14.1%)	655 (15.2%)	
Mode of HIV transmission, n (%)					
HS	2773 (34.6%)	590 (38.2%)	816 (37.7%)	1367 (31.7%)	< 0.001
PWID	1131 (14.1%)	509 (33.0%)	265 (12.2%)	357 (8.3%)	
MSM	3528 (44.0%)	342 (22.2%)	942 (43.5%)	2244 (52.0%)	
other/unknown	591 (7.4%)	102 (6.6%)	143 (6.6%)	346 (8.0%)	
Education					
Elementary school	362 (4.5%)	162 (10.5%)	99 (4.6%)	101 (2.3%)	< 0.001
Junior high school	1803 (22.5%)	596 (38.6%)	528 (24.4%)	679 (15.7%)	
High school	2576 (32.1%)	423 (27.4%)	708 (32.7%)	1445 (33.5%)	
University	920 (11.5%)	101 (6.6%)	217 (10.0%)	602 (14.0%)	
Missing data	2362 (29.4%)	261 (16.9%)	614 (28.4%)	1487 (34.5%)	
Employement					
Full time worker	3575 (44.6%)	703 (45.6%)	1100 (50.8%)	1772 (41.1%)	< 0.001
Unemployed	910 (11.3%)	307 (19.9%)	217 (10.0%)	386 (9.0%)	
Self employed	1231 (15.3%)	265 (17.2%)	304 (14.0%)	662 (15.4%)	
Temporary employed	192 (2.4%)	75 (4.9%)	45 (2.1%)	72 (1.7%)	
Student	296 (3.7%)	29 (1.9%)	75 (3.5%)	192 (4.5%)	
Retired	256 (3.2%)	40 (2.6%)	96 (4.4%)	120 (2.8%)	
Housewife	233 (2.9%)	103 (6.7%)	72 (3.3%)	58 (1.3%)	
Other/unknown	204 (2.5%)	18 (1.2%)	40 (1.9%)	146 (3.4%)	
Missing data	1126 (14.0%)	3 (0.2%)	217 (10.0%)	906 (21.0%)	
Residency area					
Northern Italy	4540 (56.6%)	763 (49.5%)	1226 (56.6%)	2551 (59.1%)	< 0.001
Central Italy	2678 (33.4%)	517 (33.5%)	769 (35.5%)	1392 (32.3%)	
Southern Italy	805 (10.0%)	263 (17.0%)	171 (7.9%)	371 (8.6%)	
Smokers					
No	3589 (44.7%	532 (34.5%)	1040 (48.0%)	2017 (46.8%)	< 0.001
Yes	3636 (45.3%)	787 (51.0%)	990 (45.7%)	1859 (43.1%)	
Not known	798 (10.0%)	224 (14.5%)	136 (6.3%)	438 (10.2%)	
CDC stage					
C	714 (8.9%)	199 (12.9%)	219 (10.1%)	296 (6.9%)	< 0.001
A/B	7309 (91.1%)	1344 (87.1%)	1947 (89.9%)	4018 (93.1%)	
Pre treatment CD4 cell/mmc					
< =200	1630 (20.3%)	404 (26.2%)	508 (23.5%)	718 (16.6%)	< 0.001
201–350	1952 (24.3%)	516 (33.4%)	699 (32.3%)	737 (17.1%)	
351–500	1761 (22.0%)	251 (16.3%)	473 (21.8%)	1037 (24.0%)	
500-max	1913 (23.8%)	308 (20.0%)	291 (13.4%)	1314 (30.5%)	
Missing data	767 (9.6%)	64 (4.2%)	195 (9.0%)	508 (11.8%)	
Pre treatment log10 HIV RNA					
< 4	1844 (23.0%)	385 (25.0%)	427 (19.7%)	1032 (23.9%)	0.012
4–4.7	1961 (24.4%)	379 (24.6%)	524 (24.2%)	1058 (24.5%)	
4.8–5.2	1644 (20.5%)	363 (23.5%)	478 (22.1%)	803 (18.6%)	
5.2+	1590 (19.8%)	316 (20.5%)	488 (22.5%)	786 (18.2%)	
Missing data	984 (12.3%)	100 (6.5%)	249 (11.5%)	635 (14.7%)	
Pregnancy status					
Not pregnant	7715 (96.2%)	1517 (98.3%)	2109 (97.4%)	4089 (94.8%)	< 0.001
Pregnant	30 (0.4%)	5 (0.3%)	7 (0.3%)	18 (0.4%)	
Missing data	278 (3.5%)	21 (1.4%)	50 (2.3%)	207 (4.8%)	

**Fig. 1:**
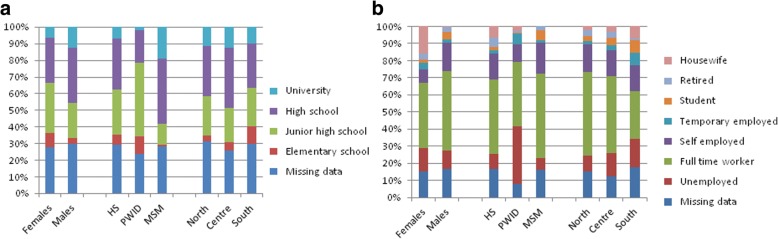
Distribution of education level (**a**) and occupational status (**b**) according to gender, mode of HIV transmission and area of residency

The distribution of educational level and occupational status according to gender, mode of HIV transmission and area of residency are shown in Fig. 1. Women, PWID and residents in Southern Italy presented the lowest levels of education and the highest rate of unemployment compared to other groups.

The median CD4 cell count at time of enrolment in the ICONA cohort according to the investigated socio-demographic variables is summarized in Table 2 In particular, women, people who acquired the HIV infection through heterosexual contacts, older individuals, and housewives, were more at risk of late presentation. Moreover, the risk of late presentation decreased with increasing educational level.

**Table 2:** Median CD4 cell count at time of enrolment in the ICONA cohort and at time of ART initiation according to socio-demographic characteristics

	CD4 at enrollment			CD4 at time of ART initiation		
	Median	IQR	*p*	Median	IQR	*p*
	n/mmc	n/mmc		n/mmc	n/mmc	
Gender						
Male	439	260–621	0.453	360	223–520	< 0.001
Female	448	263–635		325	205–479	
Age (years)						
< 30	511	364–696	< 0.001	390	273–563	< 0.001
30–40	440	269–622		358	225–514	
40–50	377	188–554		323	173–483	
> 50	283	108–500		273	105–422	
Mode of HIV transmission						
HS	385	191–580	< 0.001	310	171–460	< 0.001
PWID	482	305–685		342	222–530	
MSM	471	310–643		390	267–550	
Residency area						
North	499	336–694	< 0.001	333	232–460	< 0.001
Centre	443	270–634		302	193–436	
South	432	248–646		330	205–516	
Education						
Elementary school	384	179–606	< 0.001	288	156–430	< 0.001
Junior high school	437	232–624		320	197–481	
High school	438	261–613		353	224–505	
University	464	299–637		393	272–569	
Employement						
Unemployed	439	264–644	< 0.001	342	216–523	< 0.001
Full time worker	450	279–626		351	225–506	
Self employed	437	249–621		351	222–510	
Temporary employed	467	320–650		340	220–506	
Student	540	385–700		443	325–648	
Retired	326	131–534		274	121–440	
Housewife	390	157–590		302	122–427	
Year of ART initiation/last observation						
2002–2006	465	278–661	< 0.001	292	185–448	< 0.001
2007–2011	385	232–554		305	196–409	
2012–2016	460	270–637		414	262–574	

The number of lost to follow-up patients according to the main study variables is reported in Table 3; female gender, younger age, PWID, low educational level and unemployment were associated with a higher drop-out rate.

**Table 3:** Drop-out rate according to socio-demographic characteristics

	N° of lost to follow-up	*P*
Gender		
Male	1373 (20.8%)	< 0.001
Female	423 (29.5%)	
Age at first positive anti-HIV test		
< 30	797 (30.9%)	< 0.001
30–40	566 (21.4%)	
40–50	258 (15.1%)	
> 50	168 (15.6%)	
Mode of HIV transmission		
HS	571 (20.6%)	< 0.001
PWID	552 (48.8%)	
MSM	530 (15.0%)	
other/unknown	143 (24.2%)	
Residency area		
North	929 (20.5%)	< 0.001
Centre	560 (20.9%)	
South	307 (38.1%)	
Education		
Elementary school	141 (39.0%)	< 0.001
Junior high school	574 (31.8%)	
High school	484 (18.8%)	
University	129 (14.0%)	
Missing	468 (19.8%)	
Employment		
Unemployed	755 (21.1%)	< 0.001
Full time worker	347 (38.1%)	
Self employed	264 (21.4%)	
Temporary employed	72 (37.5%)	
Student	67 (22.6%)	
Retired	50 (19.5%)	
Housewife	94 (40.3%)	
other/unknown	33 (16.2%)	
missing data	114 (10.1%)	

### Time to ART initiation

A total of 6214 cohort participants (77.5%) started ART during the study period after a median time to ART initiation of 0.6 years (IQR 0.1–3.7). The median of years from first HIV test and cART initiation decrease over time [2002–2006 = 3.3 years (0.2–9.4); 2007–2011 = 1.0 years (0.1–3.9); 2012–2016 = 0.2 years (0.1–2.1), *p* < 0.001].

Median CD4 cell count levels at time of ART initiation are also showed in Table 2. Females, patients aged > 50 years, housewife and retired, and people with lower educational levels presented the lowest CD4 count at ART initiation compared to other groups.

**Fig. 2:**
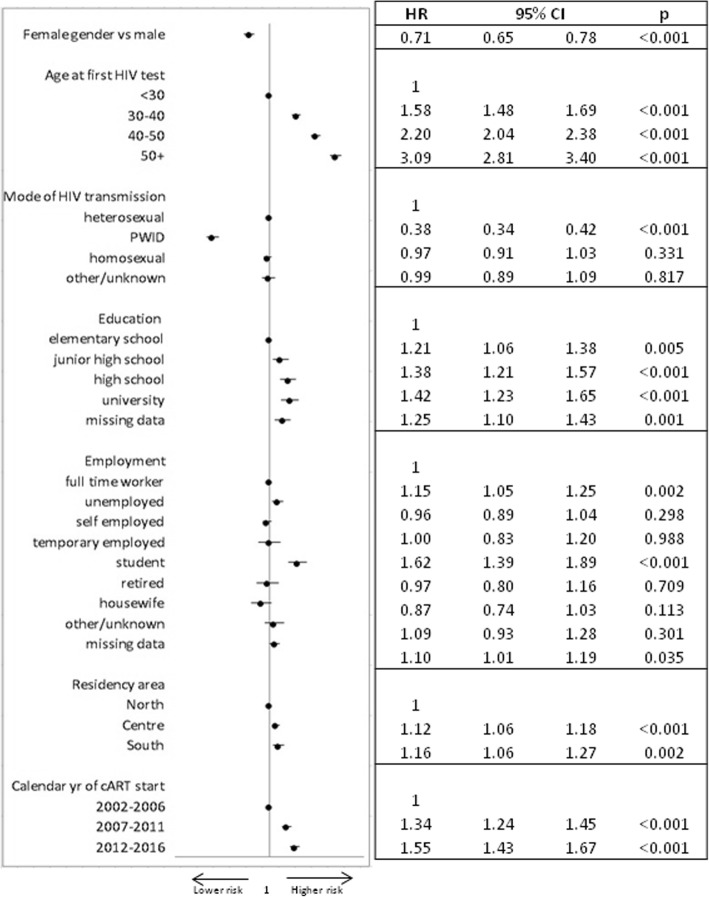
Multivariable model of factors associated with time to cART start (adjusted for CD4 count, viral load, pregnancy status, smoking)

Factors associated with the probability of ART initiation by multivariable analysis are reported in Fig. 2: females vs. males, PWID compared to HS showed a reduced probability of starting therapy. On the other hand, unemployed people and students compared to full time workers, older people, people with higher educational levels, and residents in the Central and Southern area of the country were more likely to initiate ART.

### Response to ART

**Fig. 3:**
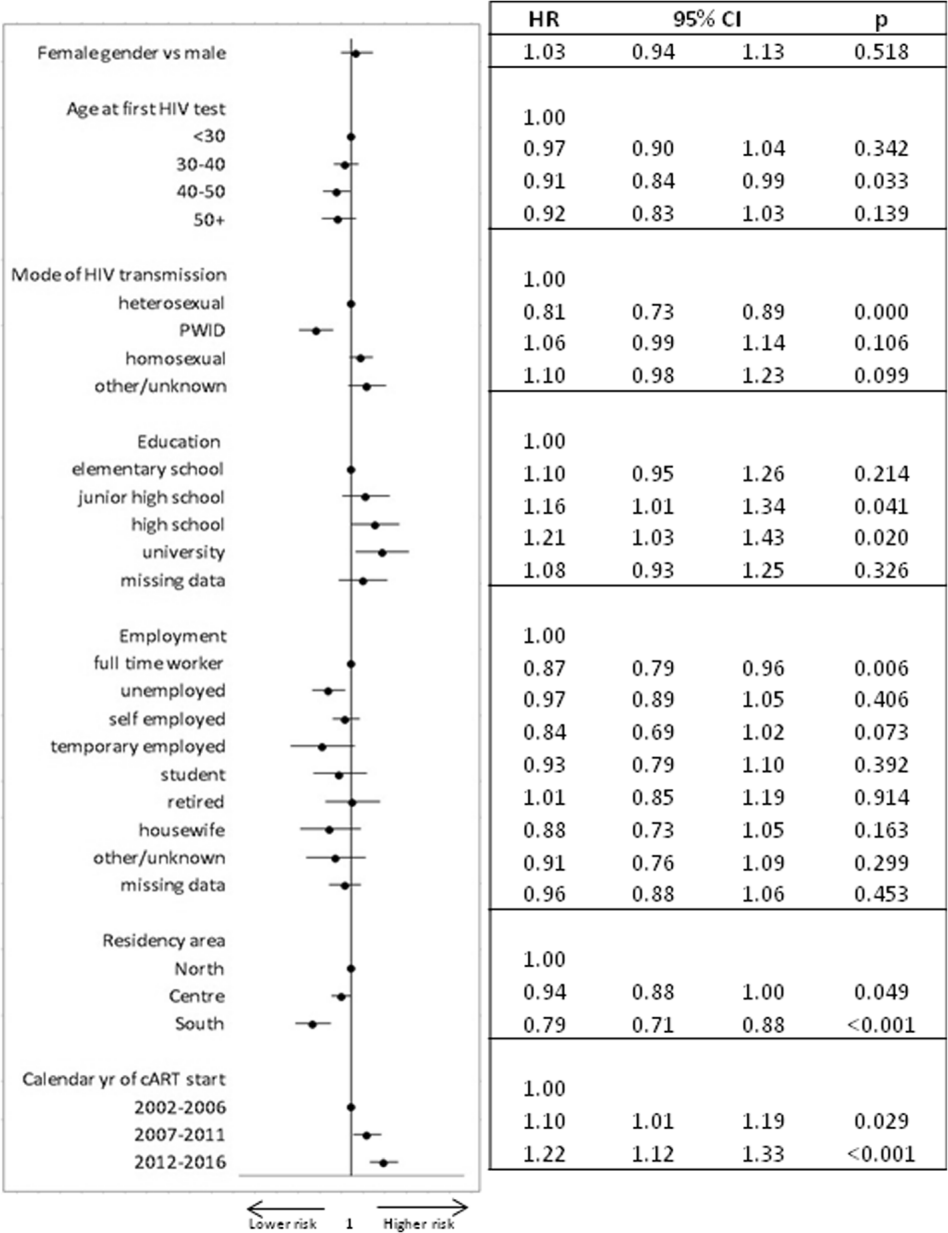
Multivariable model of factors associated with virological response (HIV RNA < 50) (adjusted for CD4 count, viral load, pregnancy status, smoking)

A total of 5792 patients starting ART during the study period presented at least 1 year of follow-up and were included in the analysis on the response to ART. 5111 (88.2%) cohort participants reached at least one HIV-RNA < 50 cp/mL. After adjustment for potential confounders, intravenous drug use compared to heterosexual as mode of HIV transmission, unemployment and temporary employment vs full time work, and residency in Southern and Central vs Northern Italy were associated with a lower probability of achieving HIV-RNA suppression. On the contrary, patients with higher educational levels (high school or university, ISCED 2–4) were more likely to reach an undetectable viral load than their counterparts (Fig. 3).

### ART discontinuation

**Fig. 4:**
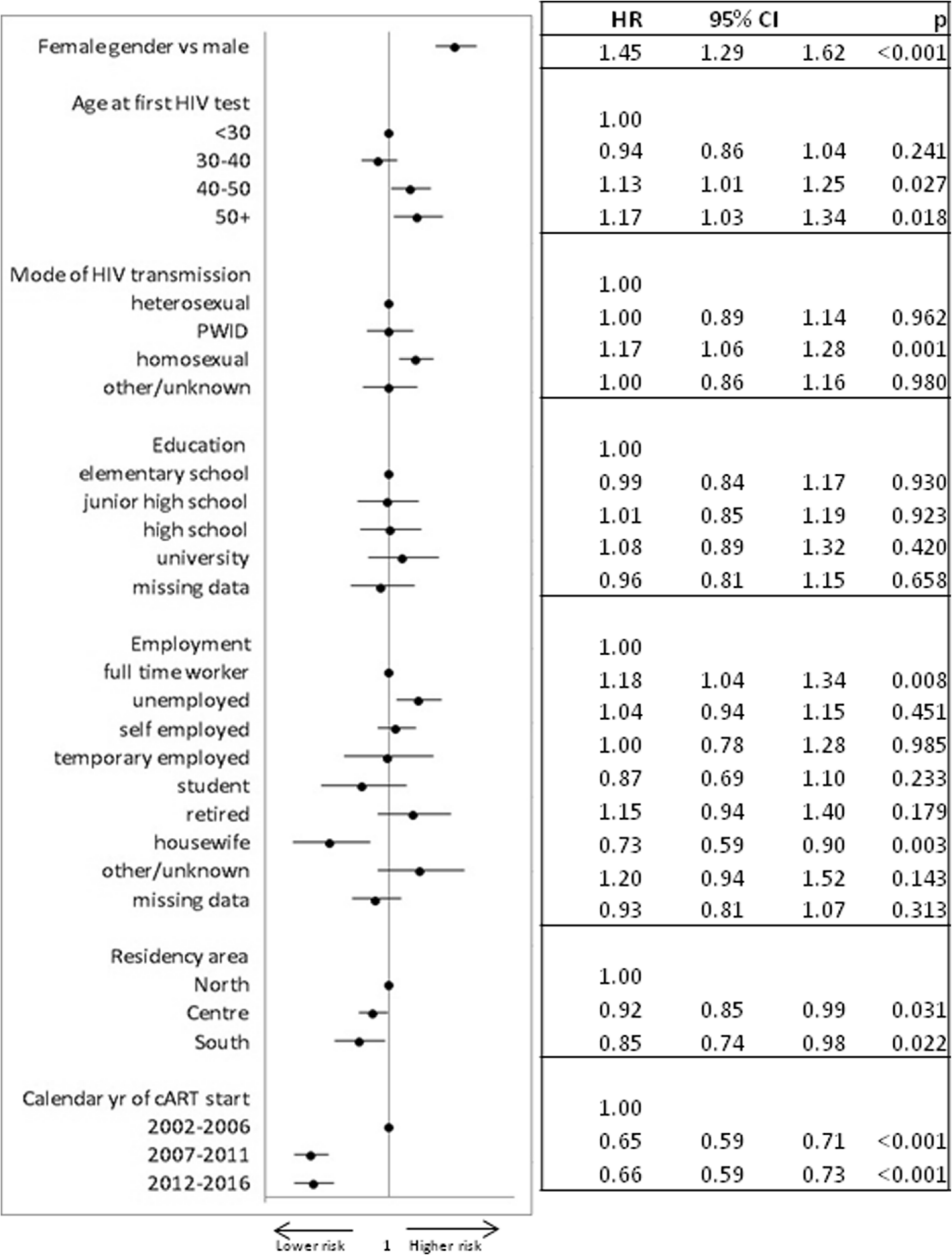
Multivariable model of factors associated with treatment discontinuation for any causes (adjusted for CD4 count, viral load, pregnancy status, smoking)

A total of 3079 out of 5792 patients (53%) changed or interrupted first line-regimen during the study period. In Fig. 4, factors associated with first-line ART discontinuation by multivariable analysis are summarized. MSM presented a higher risk of first-line ART discontinuation along with patients aged 40–50 and more than 50 years vs < 30, females vs males, unemployed people compared to full time workers. On the contrary, housewives and residents in Central and Southern Italy presented a lower rate of therapy discontinuation than other groups, while no association was observed between educational level and therapy interruption.

## Discussion

Socio-demographic factors are an important cause of health inequalities, even in high income countries adopting universal health care systems [5]. In Italy, both HIV testing and ART are free of charge for all individuals, independently on disease stage, work conditions or other economical issues. Data from the national health institute demonstrated a satisfactory framework of the HIV treatment cascade in our country, which appears to be very close to WHO targets, showing that in 2014 92% of people diagnosed with HIV were in ART treatment, 87.7% of whom with suppressed HIV viremia [6]. According to changes in international and national guidelines, which progressively suggested to start ART at higher CD4 count overtime, our data demonstrate that, for patients enrolled in the ICONA cohort, time to ART initiation significantly decreased from more than 3 years in 2002–2006 up to 0.2 years in the last 4 years. In this context, we aimed to evaluate if social determinants do still play a role in access and response to antiretroviral therapy in PLHIV.

Herein, we limited our analysis to native Italian people in order to avoid that confounding factors related to the status of migrant could influence our results, including language barriers and cultural differences. In a previous paper, however, including patients enrolled in the ICONA cohort as well, an increased risk of both delayed access to ART and virological failure was already demonstrated in migrants living with HIV [7]. Moreover, only patients enrolled in ICONA during a relative recent period (2002–2016) were included in the present study, in order to take into account exclusively modern (and highly potent) antiretroviral regimens.

The results of our analysis clearly evidenced that, even in a country with universal health care coverage as Italy, several social determinants, including female gender, PWID as a risk factor for HIV transmission, low educational level and unemployment, significantly impact both ART access and outcomes. It should be underlined that these variables are also strictly interconnected to each other: in fact, females, PWID and residents in Southern Italy also presented the lowest level of education and the highest rate of unemployment. Although the public health system is freely assured throughout the country since 1978, after a constitutional reform voted in 2001, budget and organization of health services are under regional control. Therefore, many differences in health services may exist among different Italian areas. Based on historical reasons, Northern, Central, and Southern Italy are quite different from an economic and social point of view, with a North-to-South gradient, being in general Northern regions the most industrialized areas of the nation.

First, a risk of late presentation was demonstrated for all the above mentioned categories, which was particularly evident for people aged more than 50 years, which is in line with national and international reports [6, 16]. Similarly, older patients started ART with significantly lower CD4 cell number compared to younger subjects and, in parallel, they presented significantly longer time to ART initiation since the first anti-HIV positive test. People > 50 years old had the same mode of HIV acquisition of younger people, but they may be less aware of their HIV status. Moreover, HIV testing is probably less recommended by health care providers in this group than in young adults [17]. However, old people need to be recognized as a high risk group as they present poorer immunologic response compared with younger patients, with higher mortality rates [18]. In our study, ART initiation was more likely with increasing age, even after adjustment for CD4 count. No significant association between age and achievement of viremia suppression on ART after 1 year was demonstrated. Older patients were associated with a more recent year of ART initiation, which was conversely associated with lower risk of discontinuation. From the multivariable analysis, however, after adjusting for year of ART initiation, older age was related to a higher risk of therapy discontinuation, probably due to a higher frequency of side effects and co-morbidities in this patient group. The type of drug ART regimen could also be a possible explanation, as older individuals were more frequently on a protease inhibitor-based regimen (42% compared to 25% in patients aged > 30 years, data not shown).

If female gender is a risk factor for late presentation, delayed therapy initiation and poor ART response is controversial in literature [19–21]. In fact, several aspects might contribute to confound results from different cohorts, also depending on the chosen endpoints [19]: firstly, early diagnosis of HIV infection in women is favored by screening programs for HIV during pregnancy; secondly, lower HIV-RNA levels have been observed in women compared to males [20] which could justify better response rates to ART. Finally, regarding the association of gender and mortality for PLHIV, it should be taken into account that women have an overall higher life expectancy compared to men, at least in Western countries. Moreover, in PLHIV, a lower incidence of non-AIDS events in women compared to males has been described [22]. In our study, after controlling for other variables including pregnancy status and HIV viremia levels, females resulted more at risk of delayed ART initiation and therapy discontinuation, even if no differences in response rates to the first year of ART were evidenced. The status of housewife, however, was not associated with the same endpoints in our analysis; on the contrary, they presented a lower rate of therapy discontinuation. No data regarding this association in literature are available to our knowledge.

Among the different risk group categories for HIV acquisition, as expected, people who inject drugs presented a delay in initiation of ART and inferior virological response to therapy; our results are in agreement with the paper of Lesko et al. [13] who demonstrated as PWID, even in recent years, spent a significantly larger amount of time not on ART compared with non-PWID or, when on ART, not virally suppressed, thus greatly contributing to HIV transmission. On the contrary, MSM, who became the most represented group in the last years (52%), initiated ART at significantly higher CD4 levels than other risk groups; however, MSM resulted more frequently at risk of ART change / interruption compared to other groups.

The economic status of PLHIV is always hard to be determined. Educational level and occupational status, however, can be considered good proxies of such condition [5]. We demonstrated that the educational level has a crucial role of in influencing both access and response to ART in our country. This finding extend previous observations in Italy [23] and in other European countries [24–26], and are in agreement with recent multi-cohort data from the COHERE [10, 11]. In addition, the relationship between occupational status and ART access and outcomes was also analyzed. Very few studies [12, 27] have addressed this point to date, as this information is not registered in the most part of the cohorts enrolling HIV patients. In France [27], a global indicator of social vulnerability including education, employment, and housing stability and comfort, after adjustment for lifestyle factors, psychosocial characteristics (depression, drug using) and known biomedical factors, was associated with mortality in PLHIV. Moreover, unemployment was associated with a reduced sustained virological suppression (e.g. undetectable viral load for ≥6 months) [12]. In our analysis, unemployment was associated with all three endpoints (a longer time to ART initiation, lower chances to reach a suppressed HIV viremia, and higher risk of discontinuation). It must be underlined that work conditions, although considered an excellent proxy of economic status, may change during patient’s life and cannot be considered as an independent variable as a poor health status could also cause job loss. However, as only the first year ART response was investigated in the present study, and the occupational status was registered at entry in the cohort, our results can be considered reliable.

Among lifestyle factors, smoking was not associated with any of the study endpoints in our analysis [14]. All results were adjusted for smoking and pregnancy status. However, other behavioral and clinical factors, including alcoholism or depression, were not targeted in our analysis but could have indirectly influenced our results. In particular, the inclusion of mental health outcomes as part of the ICONA database in the future would be suitable. A high percentage of missing data regarding educational level could have introduced some biases, representing an important limitation of our study. A major limitation is also the lack of information regarding the presence of transgender people in our cohort. In fact, even if sexual orientation and gender identity should be reported the ICONA database, no transgender cohort participants were registered among patients fulfilling inclusion criteria in our study, which seems to suggest that this information was under-reported by clinicians [28, 29].

## Conclusions

In conclusions, notwithstanding median time to ART start decreased from 3.3 to 0.2 years from 2002 to 2016, socio-demographic factors still contribute to important disparities in ART initiation, outcome and durability in PLHIV from Italy. Therefore, a deeper evaluation of socio-economic determinants, possibly using a standardized approach, is needed on one side; on the other side, welfare interventions aiming to reduce the social inequality for fragile populations such as PWID, women, old people, patients with low educational level and unemployed, along with migrants, would indirectly improve the cascade of care for PLHIV. Moreover, it should be underlined that relatively few complete national data regarding continuum of care are available in Italy as in other countries, with wide variation in reporting procedures [30]. Nowadays it becomes more and more important to use consistent and accurate methods for tracking progress towards the achievement of WHO 90–90-90 targets.

## Abbreviations

ART: Antiretroviral Therapy
CDC: Center for Disease Control
HIV: Human Immunodeficiency Virus
HS: Heterosexual
ICONA: Italian Cohort Naïve Antiretrovirals
IQR: Interquartile range
IRB: Institutional Review Board
ISCED: International Standard Classification of Education
MSM: Men who have sex with men
PLHIV: People Living With HIV
PWID: People who inject drugs
UNAIDS: Joint United Nations Programme on HIV/AIDS
UNESCO: United Nations Educational, Scientific and Cultural Organization
WHO: World Health Organization
